# The Qualified Dentist

**Published:** 1897-09-04

**Authors:** 


### The Qualified Dentist.

Within the last twenty years the status of the dental profession has been enormously raised. This has been due to the recognition of dentistry by the medical licensing bodies and to the provision that has been made for registering the dental qualification. Until comparatively recently anyone that liked could not only set up as a dentist but get the public to entrust their teeth to him. A forceps and a placard inscribed "Teeth extracted here" were all that was necessary in the way of stock in trade. As a matter of fact, it was not until the authorities stepped in and established the qualification that the public was in a position to protect itself. There was no means of distinguishing between the competent and the incompetent practitioner.

All this has been changed. The jaws of the people are no longer at the mercy of ignorant bungling. The unqualified dentist is going the way of the quack doctor, and although something yet remains to be done in the way of educating the public as to the difference between the dentist who is accredited and the dentist who is not, there is little doubt that in a few years the value of the qualification will be universally recognised.

It is not very much easier to become a dentist than to become a doctor. The course of study prescribed for the dental diploma by the Royal College of Surgeons is one of four years. The subjects of preliminary examination in general education and in science are the same for the dental as for the medical student; and before the qualification can be obtained the student must profess not only the special knowledge of the teeth and their diseases, but "general anatomy and physiology, general surgery and pathology." It is noteworthy that in connection with the dental course the system of apprenticeship that once obtained in medicine is recognised. Candidates for the diploma must produce certificates of having received three years' instruction in mechanical dentistry from a registered practitioner.

At the present moment the dental is one of the most desirable of the professions. It is not so full as medicine is. The diseases to which the dentist ministers are always with us, nor can it be said that they are likely to become less prevalent. Moreover, the qualified dentist has now almost the whole field to himself. On the one hand, the unqualified dentist has been ousted; on the other, medical men have almost entirely ceased to treat diseases of the teeth; they invariably refer patients who need treatment of this kind to the dentist. Thus it is that it is quite worth the while of the young man who is choosing a career to consider whether he should not become a dentist.

The alternative—doctor or dentist—is not a necessary one. One may well be both; many are. With a little scheming the student may take out both courses at the same time. Many who set out to become dentists have ended by becoming doctors as well, and there are cases of full-fledged physicians and surgeons who have found it to their interest to take the dental diploma as well. It is obvious that it strengthens the position of the dentist very much to hold a medical as well as a dental qualification; and as the course of study is up to a certain point the same, it has become quite common for the student to go forward to both qualifications.

There are three large dental schools in London—the Dental Hospital in Leicester Square, the National Dental Hospital, and the Dental School at Guy's Hospital. In the provinces, Birmingham, Liverpool, Bristol, and Manchester make provision for instruction in dentistry; so do Edinburgh, Glasgow, and Dublin.

